# Supplementary material for: Factors Associated with Polyp Detection Rate in European Colonoscopy Practice: Findings of The European Colonoscopy Quality Investigation (ECQI) Group
Source: Int J Environ Res Public Health. 2022 Mar 13;19(6):3388. doi: 10.3390/ijerph19063388 (PMC8954761; doi:10.3390/ijerph19063388)
Supplement: Supplementary file 1 [file ijerph-19-03388-s001.zip › ijerph-1577673-supplementary materials.pdf]

PROCEDURE

ECQI
EUROPEAN COLONOSCOPY QUALITY INVESTIGATION GROUP

Patient Information

1. Year of birth:

[ ] YYYY

2. Gender: ***(Please select one)***

☐ Male ☐ Female

3. Height:

[ ] cm

4. Weight:

[ ] kg

5. BMI:

[ ] kg/m²

6. Type: ***(Please select one)***

☐ In-patient ☐ Out-patient

Pre-Procedure

7. Patient referred by: ***(Please select one)***

☐ GP
☐ Own speciality
☐ Screening programme
☐ Self-referred
☐ Other *(Please state)*

[ ]

8. Reason for procedure: ***(Please select one)***

- [ ] Clinical signs and symptoms *(Please select all that apply)*
  - [ ] Altered bowel
  - [ ] Pain
  - [ ] Rectal bleeding
  - [ ] Other *(Please state)*
- [ ] Follow-up
- [ ] Following positive screening test
- [ ] Previous unsuccessful procedure
- [ ] Screening due to familial risk
- [ ] Screening without pre-screening test
- [ ] Other *(Please state)*

9. Bowel preparation used? ***(Please select one)***

- [ ] Yes *(Please answer the following)* - [ ] No

Type, including additional products: ***(Please select all that apply)***

- [ ] 2 litre PEG and ascorbate
- [ ] 4 litre PEG
- [ ] Bisacodyl
- [ ] Enema
- [ ] Sodium phosphate
- [ ] Sodium picosulphate and magnesium citrate
- [ ] Tri-sulphate
- [ ] Other *(Please state)*

Dosing regimen: ***(Please select one)***

- [ ] Evening
- [ ] Same day
- [ ] Split
- [ ] Other *(Please state)*

Did the patient follow the bowel preparation instructions? ***(Please select one)***

- [ ] Yes - [ ] No

How much bowel preparation was consumed? *(Please select one)*

- ☐ 0 - 25%
- ☐ 25 - 50%
- ☐ 50 - 75%
- ☐ 75 - 99 %
- ☐ 100%

How much fluid was consumed in total, including additional products? *(Please select one)*

- ☐ 0 - 1 litre
- ☐ 1 - 3 litres
- ☐ 3 - 6 litres
- ☐ Over 6 litres

Time period between last intake of bowel preparation and procedure:

[ ] hours

10. Time of colonoscopy:

[ ] HH:MM

11. Patient had a total colonoscopy in the last 5 years? *(Please select one)*

☐ Yes *(Please answer the following)* ☐ No

Date of last total colonoscopy:

[ ] MM [ ] YYYY

Procedure

12. Sedation-related medications administered? *(Please select one)*

☐ Yes *(Please select all that apply)* ☐ No

- ☐ Entonox
- ☐ General Anaesthesia
- ☐ Midazolam
- ☐ Opiates
- ☐ Propofol
- ☐ Other *(Please state)*

[ ]

13. Procedure-related medications administered? *(Please select one)*

- ☐ Yes *(Please select all that apply)* ☐ No
  - ☐ Buscopan
  - ☐ Glucagon
  - ☐ Other *(Please state)*

14. Medication administered by: *(Please select all that apply)*

- ☐ Anaesthetist
- ☐ Endoscopist
- ☐ Qualified nurse
- ☐ Second doctor
- ☐ Other *(Please state)*

15. Chromoendoscopy used? *(Please select one)*

- ☐ Yes *(Please select all that apply)* ☐ No
  - ☐ Localised (digital)
  - ☐ Localised (dye)
  - ☐ Pan (digital)
  - ☐ Pan (dye)

16. High definition equipment used? *(Please select one)*

- ☐ Yes *(Please select all that apply)* ☐ No ☐ Don't know
  - ☐ Scope
  - ☐ Monitor/Screen

17. Assistive technology used? *(Please select one)*

- ☐ Yes *(Please select all that apply)* ☐ No
  - ☐ Cap-assisted
  - ☐ Endocuff-assisted
  - ☐ Scope guide
  - ☐ X-ray
  - ☐ Other *(Please state)*

18. Intended endpoint: *(Please select one)*

- ☐ Anastomosis
- ☐ Caecum
- ☐ Terminal ileum/Neo terminal ileum

19. Endpoint photo documented? *(Please select one)*

- ☐ Yes ☐ No

20. Intended endpoint reached? *(Please select one)*

- ☐ Yes ☐ No *(Please select one)*
  - ☐ Insufficient preparation
  - ☐ Pain
  - ☐ Pathology encountered
  - ☐ Stricture
  - ☐ Technically difficult
  - ☐ Unsafe procedure
  - ☐ Other *(Please state)*

21. Retraction time recorded? *(Please select one)*

- ☐ Yes *(Please state time)* ☐ No
  - minutes

22. Abnormal endoscopic finding? *(Please select one)*

- ☐ Yes *(Please select all that apply)* ☐ No
  - ☐ Cancer
  - ☐ Diverticulae
  - ☐ Inflammation
  - ☐ Polyps
  - ☐ Other *(Please state)*

Right Colon Segment Classification

23. Cleansing quality: *(Please select one)* *

- ☐ Excellent
- ☐ Good
- ☐ Poor
- ☐ Inadequate

24. Polyps detected? ***(Please select one)***

☐ Yes *(Please answer the following)* ☐ No

Number of polyps:

[ ]

Type of polyps: ***(Please select all that apply)***

☐ Protruded lesion: Pedunculated polyp (Ip)
☐ Protruded lesion: Subpedunculated polyp (Isp)
☐ Protruded lesion: Sessile polyp (Is)
☐ Flat elevated lesion: Flat elevation of mucosa (0-IIa)
☐ Flat elevated lesion: Flat elevation with central depression (0-IIa/c)
☐ Flat lesion: Flat mucosal change (0-IIb)
☐ Flat lesion: Mucosal depression (0-IIc)
☐ Flat lesion: Mucosal depression with raised edge (0-IIc/IIa)

Size of largest polyp:

[ ] mm

Were you able to characterise the endoscopic appearance of highest grade polyp? ***(Please select one)***

☐ Yes *(Please select one)* ☐ No

☐ Adenoma
☐ Hyperplastic
☐ Malignant
☐ Sessile serrated

Transverse Colon Segment Classification

25. Cleansing quality: ***(Please select one)*** *****

☐ Excellent
☐ Good
☐ Poor
☐ Inadequate

26. Polyps detected? *(Please select one)*

☐ Yes *(Please answer the following)* ☐ No

Number of polyps:

Type of polyps: *(Please select all that apply)*

☐ Protruded lesion: Pedunculated polyp (Ip)
☐ Protruded lesion: Subpedunculated polyp (Isp)
☐ Protruded lesion: Sessile polyp (Is)
☐ Flat elevated lesion: Flat elevation of mucosa (0-IIa)
☐ Flat elevated lesion: Flat elevation with central depression (0-IIa/c)
☐ Flat lesion: Flat mucosal change (0-IIb)
☐ Flat lesion: Mucosal depression (0-IIc)
☐ Flat lesion: Mucosal depression with raised edge (0-IIc/IIa)

Size of largest polyp:

mm

Were you able to characterise the endoscopic appearance of highest grade polyp? *(Please select one)*

☐ Yes *(Please select one)* ☐ No

- ☐ Adenoma
- ☐ Hyperplastic
- ☐ Malignant
- ☐ Sessile serrated

Left Colon Segment Classification

27. Cleansing quality: *(Please select one)* *

☐ Excellent
☐ Good
☐ Poor
☐ Inadequate

28. Polyps detected? ***(Please select one)***

☐ Yes *(Please answer the following)* ☐ No

Number of polyps:

[ ]

Type of polyps: ***(Please select all that apply)***

☐ Protruded lesion: Pedunculated polyp (Ip)
☐ Protruded lesion: Subpedunculated polyp (Isp)
☐ Protruded lesion: Sessile polyp (Is)
☐ Flat elevated lesion: Flat elevation of mucosa (0-IIa)
☐ Flat elevated lesion: Flat elevation with central depression (0-IIa/c)
☐ Flat lesion: Flat mucosal change (0-IIb)
☐ Flat lesion: Mucosal depression (0-IIc)
☐ Flat lesion: Mucosal depression with raised edge (0-IIc/IIa)

Size of largest polyp:

[ ] mm

Were you able to characterise the endoscopic appearance of highest grade polyp? ***(Please select one)***

☐ Yes *(Please select one)* ☐ No

- ☐ Adenoma
- ☐ Hyperplastic
- ☐ Malignant
- ☐ Sessile serrated

*** Boston Bowel Preparation Scale (BBPS)**
Questions 23, 25, and 27 relate to the BBPS.

Excellent (3): Entire mucosa of colon segment seen well with no residual staining, small fragments of stool or opaque liquid.
Good (2): Minor amount of residual staining, small fragments of stool and/or opaque liquid, but mucosa of colon segment seen well.
Poor (1): Portion of mucosa of the colon segment seen, but other areas of the colon segment not well seen due to staining, residual stool and/or opaque liquid.
Inadequate (0): Unprepared colon segment with mucosa not seen due to solid stool that cannot be cleared.

The calculated total score and grade will be displayed following segment classification.

Procedure

29. Bowel cleansing considered acceptable for the purpose of the procedure? *(Please select one)*

- ☐ Yes ☐ No

30. Endoscopic intervention? *(Please select one)*

- ☐ Yes *(Please select all that apply)* ☐ No
  - ☐ Dilation of stenosis
  - ☐ Endoscopic mucosal resection
  - ☐ Endoscopic submucosal dissection
  - ☐ Haemostasis
  - ☐ Perforation repair by endoscopist
  - ☐ Polypectomy (complete)
  - ☐ Polypectomy (incomplete)
  - ☐ Stent insertion
  - ☐ Tattooing
  - ☐ Other *(Please state)*

31. Immediate complications? *(Please select one)*

- ☐ Yes *(Please select all that apply)* ☐ No
  - ☐ Bleeding requires admission
  - ☐ Perforation *(Please select one)*
    - ☐ Repaired
    - ☐ Not repaired
  - ☐ Sedation-related
  - ☐ Severe pain
  - ☐ Other *(Please state)*

Post Procedure

32. Non-routine (immediate) repeat procedure required? ***(Please select one)***

- ☐ Yes *(Please select one)* ☐ No
  - ☐ Further treatment or intervention required
  - ☐ Insufficient bowel preparation
  - ☐ Pain
  - ☐ Pathology encountered
  - ☐ Stricture
  - ☐ Technically difficult
  - ☐ Unsafe procedure
  - ☐ Other *(Please state)*

33. Patient successfully discharged, if classified as out-patient? ***(Please select one)***

☐ Yes ☐ No

Evaluation

34. Time to complete this form:

________ minutes

Important: Adverse events should be reported using the AE reporting system in the relevant country.
